# Supplementary material for: Microstructure and Electrical Conductivity of Electrospun Titanium Oxynitride Carbon Composite Nanofibers
Source: Nanomaterials (Basel). 2022 Jun 24;12(13):2177. doi: 10.3390/nano12132177 (PMC9268360; doi:10.3390/nano12132177)
Supplement: Supplementary file 1 [file nanomaterials-12-02177-s001.zip › nanomaterials-1766686-supplementary.pdf]

nanomaterials

Supplementary Material

Microstructure and Electrical Conductivity of Electrospun Titanium Oxynitride Carbon Composite Nanofibers

Gorazd Koderman Podboršek [1,2,*], Špela Zupančič [3], Rok Kaufman [4], Angelja Kjara Surca [1], Aleš Marsel [1], Andraž Pavlišič [5], Nejc Hodnik [1,2], Goran Dražić [1,2] and Marjan Bele [1,*]

[1] Department of Materials Chemistry, National Institute of Chemistry, Hajdrihova 19, SI-1000 Ljubljana, Slovenia; angelja.k.surca@ki.si (A.K.S.); ales.marsel@ki.si (A.M.); nejc.hodnik@ki.si (N.H.); goran.drazic@ki.si (G.D.)

[2] Jožef Stefan International Postgraduate School, Jamova 39, SI-1000 Ljubljana, Slovenia

[3] Department of Pharmaceutical Technology, Faculty of Pharmacy, University of Ljubljana, Aškerčeva 7, SI-1000 Ljubljana, Slovenia; spela.zupancic@ffa.uni-lj.si

[4] Faculty of Mathematics and Physics, University of Ljubljana, Jadranska 19, SI-1000 Ljubljana, Slovenia; rok.kaufman@student.fmf.uni-lj.si

[5] Department of Catalysis and Chemical Reaction Engineering, National Institute of Chemistry, Hajdrihova 19, SI-1000 Ljubljana, Slovenia; andraz.pavlisic@ki.si

* Correspondence: gorazd.kp@ki.si (G.K.P.); marjan.bele@ki.si (M.B.)

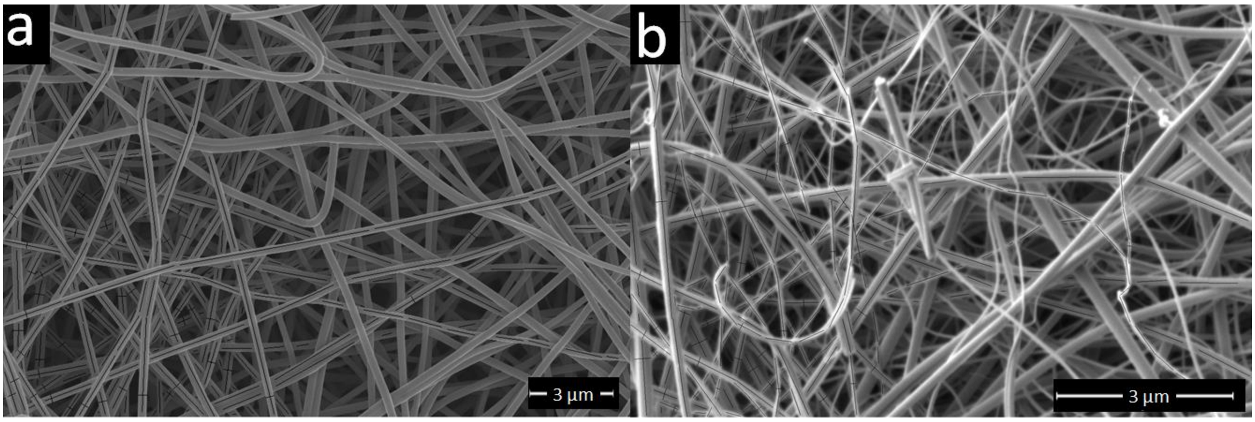

Figure S1. SEM image of TiON/C-CNF1s (**a**) and TiON/C-CNF2s (**b**) used for the diameter determination. The measurements started in the lower left corner and stopped at the 50th measurement.

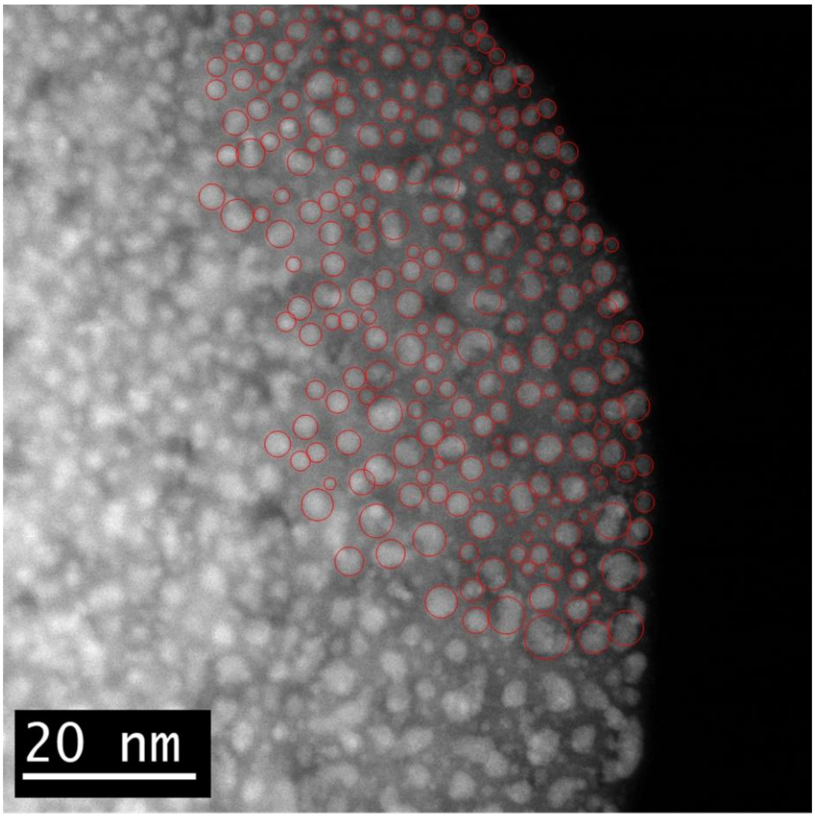

Figure S2. STEM-HAADF image of TiON nanoparticles of TiON/C-CNF2 used for the diameter determination. The measurements started in the upper right corner and stopped at the 250th measurement. Nanoparticle size was approximated with a circle.

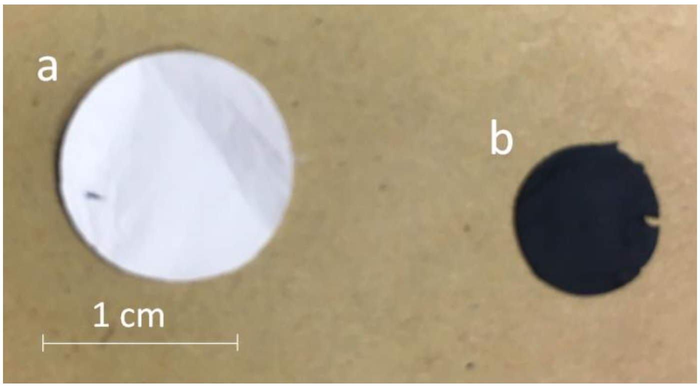

Figure S3. TiON/C-CNF1 fabric sample before nitridation (**a**) and after nitridation (**b**).

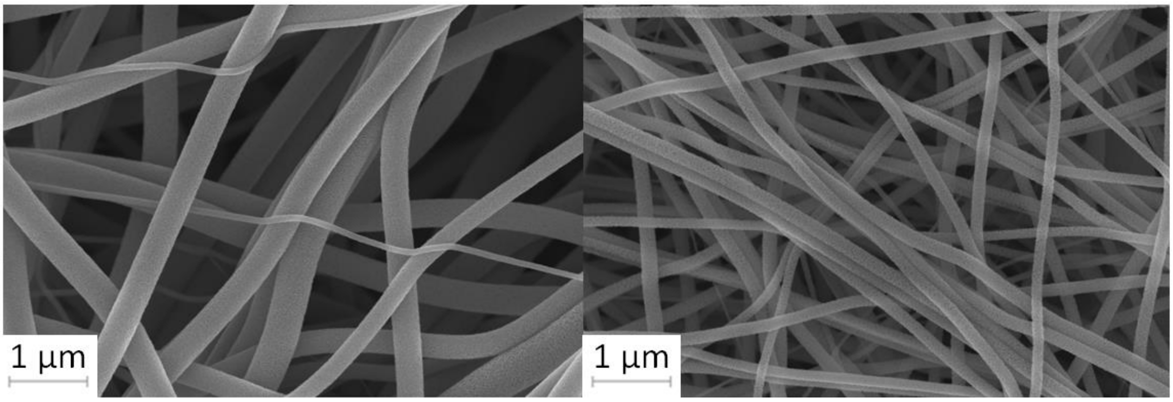

Figure S4. SEM images of contacts between TiON/C-CNF1s.

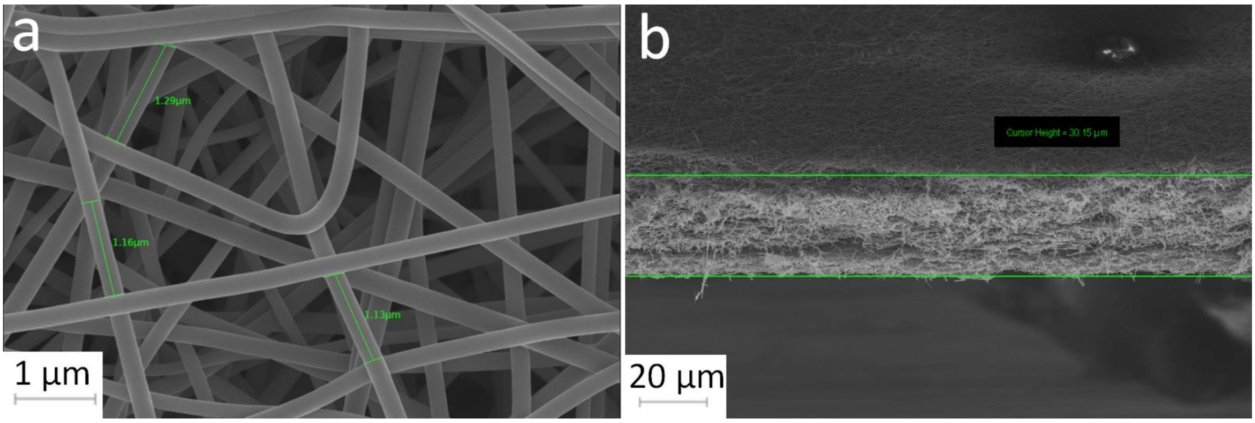

Figure S5. SEM image of TiON/C-CNF1 fabric with examples of distances between nanofiber contacts (**a**) and TiON/C-CNF1 fabric thickness (**b**).

SI 1. TGA and CHNS Elemental Analysis

The TiON/C-CNF2 fabric was analysed as bulk with TGA and CHNS analysis to obtain at. % of the elements. If we assume that only TiO_2 is left after TGA analysis, we can calculate the mass % of Ti (70.2%) in the starting sample from the end mass % in Figure S6. CHNS analysis gives us the mass % of C (5.2%), H (0.9%), and N (14%). We can assume that the rest of the mass % is from O (9.7%). From that, we can calculate at. % of the elements. The result is 33.3 at. % Ti, 9.8 at. % C, 0.2 at. % H, 22.7 at. % N, and 13.8 at. % O. The N/O at. ratio is 1.65.

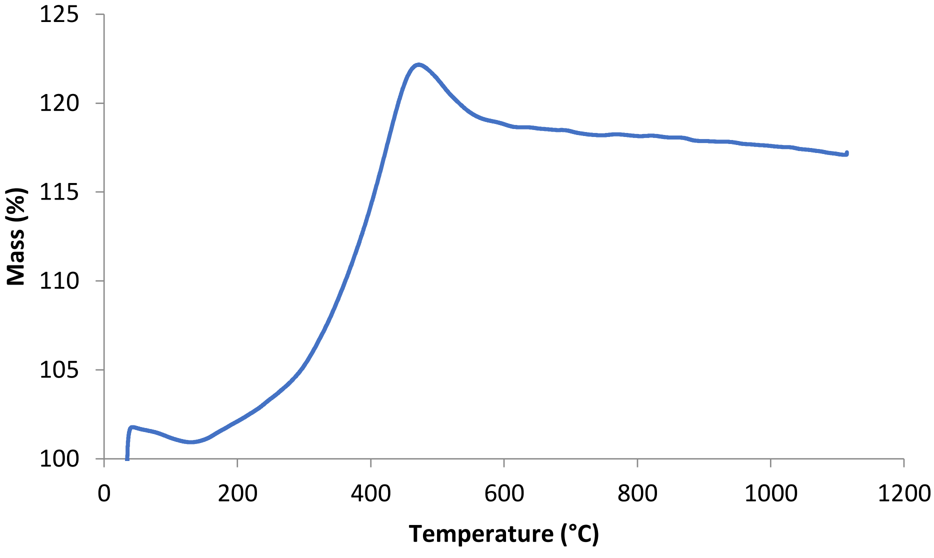

Figure S6. Thermogravimetric analysis (TGA) graph of the TiON/C-CNF2 fabric.

SI 2. Raman Spectroscopy of TiON/C-CNF2s

The Raman spectra of the TiON/C-CNF2 fabric were measured using increasing laser powers to verify the stability of the nanofibers (Figure S7). Namely, it has been reported that the formation of surface oxides may be induced by laser heating on various small-particle-sized nitride samples, including TiN [1]. Similarly, Doiron et al claimed that the existing surface amorphous TiO_2 on TiN stripes turned into anatase under 20 mW illumination for 15 min [2]. From our optical images in Figure S7 c1, d1, it is obvious that higher laser powers changed the nanofibers. The markable transformation of the nanofiber sample started with the laser power of 1.4 mW but showed the appearance of

anatase at 3.4 mW (Figure S7c,d). Namely, the bands in Figure S7 d agree well with the six modes of anatase, as determined for a single crystal by Ohsaka et al [3], which are as follows: 144 cm^{-1} (E_g), 197 cm^{-1} (E_g), 399 cm^{-1} (B_{1g}), 513 cm^{-1} (A_{1g}), 519 cm^{-1} (B_{1g}), and 639 cm^{-1} (E_g).

Accordingly, only the Raman spectra measured using lower laser powers of 0.3 mW and 0.6 mW are representative of the sample (Figure S7a,b). Both spectra have equal characteristics. The predominant bands are D and G bands of carbon at 1356 cm^{-1} and 1605 cm^{-1} that shifted slightly towards lower wavenumbers with increasing laser power. Simultaneously, the intensity of the D band increased relatively to the G band, which point to increasing disorder with increasing laser power. The presence of carbon in samples was also confirmed by SEM EDXS measurements (Figure S16).

The most interesting feature in the spectra in Figure S7a,b is composed of the broad overlapped bands below 800 cm^{-1}. This is the spectral region in which the TiN bands appear. For the above-mentioned TiN stripes, Doiron et al found the typical TiN doublet at 200 cm^{-1} and 260 cm^{-1} – 270 cm^{-1}, in addition to a broader band at ~450 cm^{-1} [2]. Other reports found these bands in the regions 180 cm^{-1} – 330 cm^{-1} (doublet) and 520 cm^{-1} – 610 cm^{-1} (broader band), depending on the preparation routes and particle size [4–6]. The bands in Figure S7a,b with peaks at 222 cm^{-1} and 538 cm^{-1} – 593 cm^{-1} resemble the double-structured shape of TiN bands (doublet and broader band). Only the low-frequency band of the doublet is visible at 222 cm^{-1} but passes over into the broad band centred at 575 cm^{-1}. This suggests that TiON was formed, which is already evident from the XRD patterns (Figure 4). Until now, we could not find any systematic investigation of Raman spectra of TiON in the literature and this topic is currently under investigation in our laboratory. The SEM EDXS results (Figure S16) confirmed the presence of Ti, N, and O, which further confirm the presence of TiON. However, the existence of a small amount of amorphous TiO_2 in nanofibers cannot be excluded only based on Raman spectra.

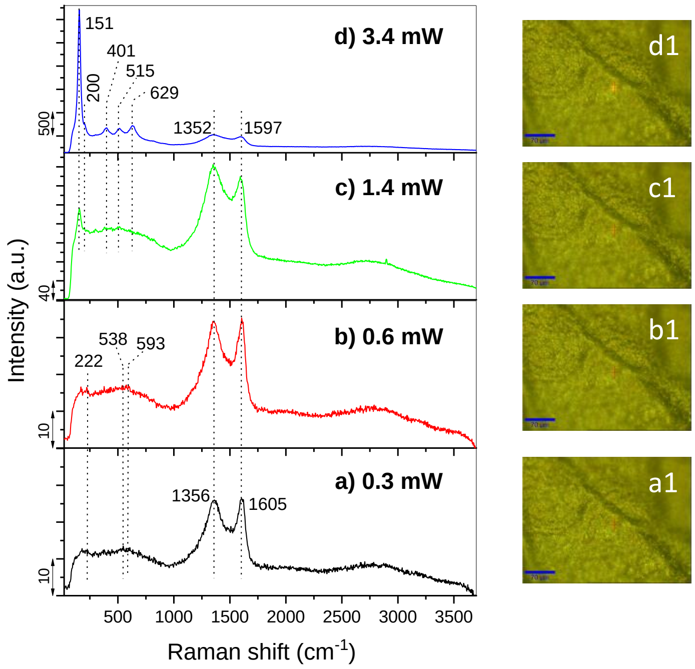

Figure S7. Raman spectra of TiON/C–CNF2s recorded on the same position using increasing laser powers of: (**a**) 0.3 mW, (**b**) 0.6 mW, (**c**) 1.4 mW, and (**d**) 3.4 mW. Besides the spectra are the optical images: (**a1**) initial - before spectrum (**a**) measurement and (**b1–d1**) after spectra (**b–d**) measurements. The red cross in the optical images indicates the measurement site.

SI 3. Simulation of STEM EDXS Spectra of TiON/C–CNF2s

As the quantitative EDXS analysis of light elements is a challenge, especially if the geometry of the sample is not ideal (flat and uniform), the STEM EDXS spectra of TiON/C-CNF2s were simulated using Monte Carlo code DTSA-II [7] to confirm the quantification results from the experimental STEM EDXS measurements (Figure S8). One experimental STEM EDXS spectrum was chosen and its quantification results were used to simulate a STEM EDXS spectrum. The atomic percentages of the experimental measurement quantification were 8.6 at. % C, 20.6 at. % N, 21.3 at. % O, and 49.5 at. % Ti. The diameter of the measured TiON/C-CNF2 was 60 nm. The % of C was increased for better fitting of the C peak, the at. % decreased but the ratio was kept the same. The at. % used for the simulated STEM EDXS spectrum were 12 at. % C, 19.85 at. % N, 20.5 at. % O, and 47.65 at. % Ti. The material was simulated as a sphere with a 60 nm diameter and a density of 3.9 g/cm^3. The resulted simulated STEM EDXS spectrum is reasonably close to the experimental STEM EDXS spectrum.

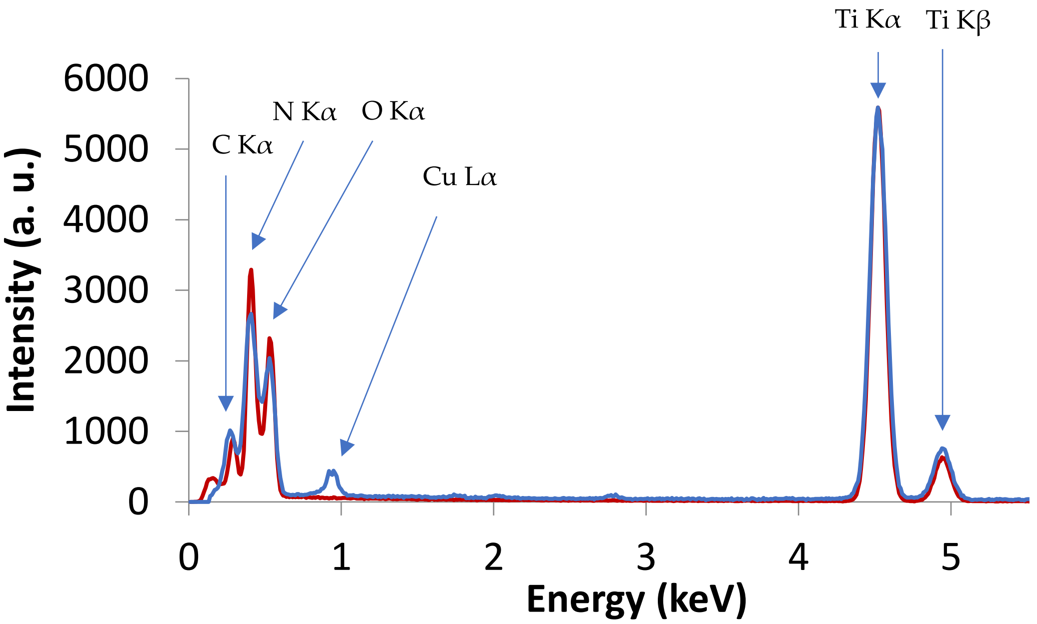

Figure S8. STEM EDXS spectrum of a TiON/C-CNF2 (blue line) and a simulated STEM EDXS spectrum of a sphere consisting of C, Ti, O, N (red line).

SI 4. N/O at. Ratio Determination with EELS

Figure S9 shows a typical EELS spectrum of a TiON/C-CNF2. The signals for N (401 eV), Ti (456 eV), and O (532 eV) are well resolved and separated, which enables us to quantify the area of the edges of all three elements and determine their atomic percentages. For N, the offset for background determination was chosen to be -43.1 eV relative to the start of the signal, with a width of 35.0 eV. The offset for the signal was chosen to be -0.1 eV and a width of 25.0 eV. For Ti, the offset for background determination was chosen to be -23.1 eV relative to the start of the signal, with a width of 20.0 eV. The offset for the signal was chosen to be -0.1 eV and a width of 25.0 eV. For O, the offset for background determination was chosen to be -28.1 eV relative to the start of the signal, with a width of 35.0 eV. The offset for the signal was chosen to be -0.1 eV and a width of 25.0 eV. The background model for all three elements was power law and was subtracted from the signal before the signal was integrated. The integration values were then calculated by the program Gatan Digital Micrograph and expressed as atomic percentage of each of the three elements. The atomic percentage of N was then divided by the atomic percentage of O to obtain the N/O at. ratio. The starting points and ranges of the back-

grounds and signals can be observed in Figure S10. The average N/O at. ratio was obtained from 4 TiON thinner nanofibers (30 nm – 75 nm diameter) from 10 locations total as an area EELS signal measurement (Figure S14). The same principle was applied for EELS mapping.

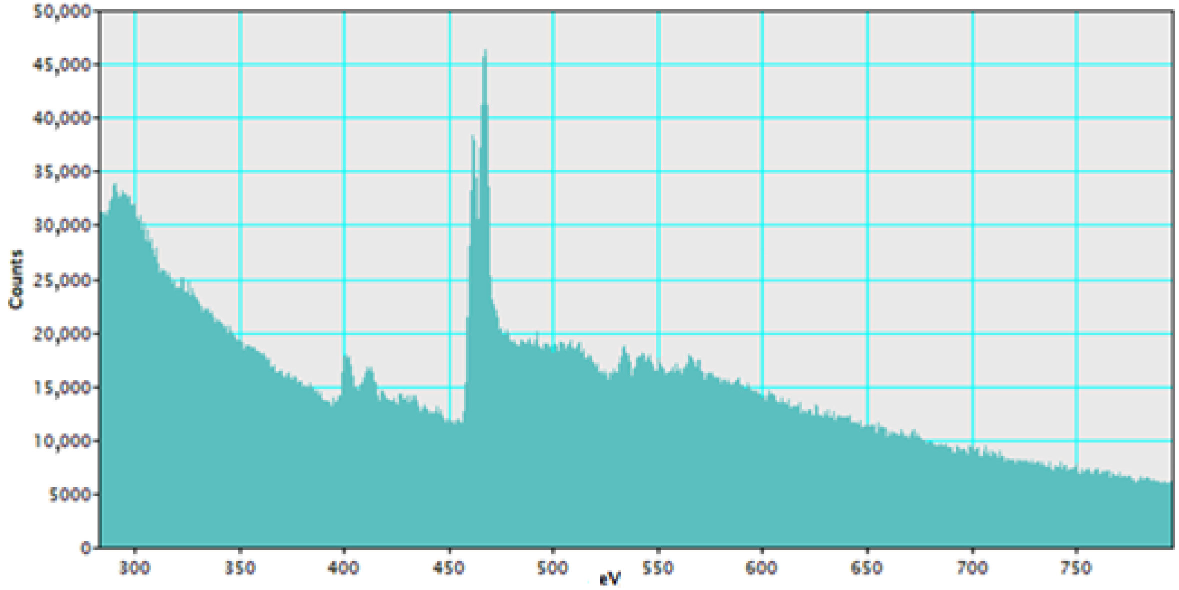

Figure S9. Typical EELS spectrum of a TiON/C–CNF2.

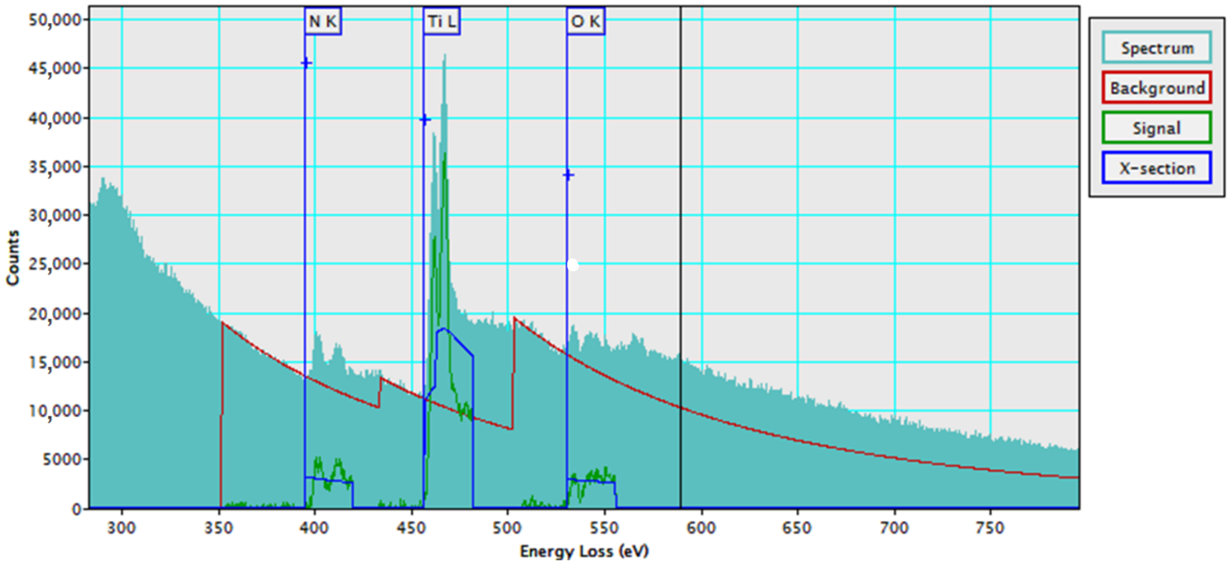

Figure S10. Typical EELS spectrum of a TiON/C–CNF2 with labelled starting points of background determination for background subtraction (red), starting points and ranges of signal integration (blue) and the resulting signal after background subtraction (green).

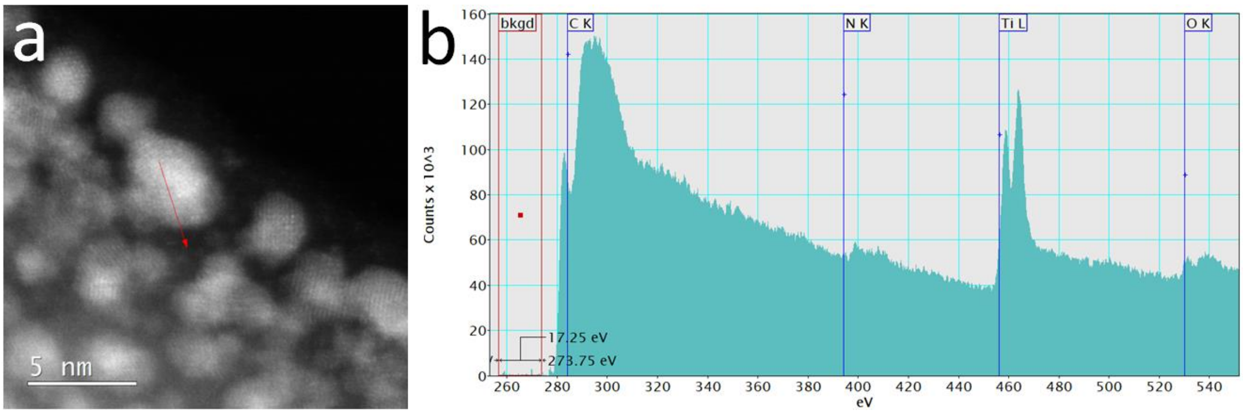

Figure S11. EELS spectrum of the carbon matrix of the TiON/C-CNF2 (**b**) with background subtracted and an STEM-HAADF image of the location where the EELS spectrum was taken ((**a**), red arrow).

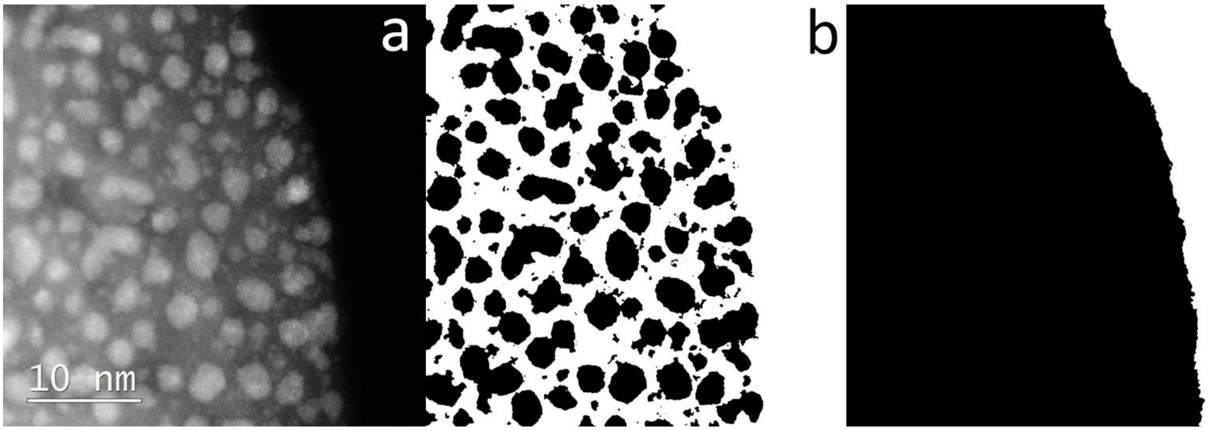

Figure S12. STEM–HAADF image of the two-phase nanocomposite TiON/C–CNF2 consisting of TiON nanoparticles and C matrix (**a**), outline of the TiON nanoparticles made with Gaussian blur, rolling ball background subtraction and thresholding (**b**), outline of the TiON/C–CNF2 area made with Gaussian blur and thresholding (**c**). Nanopore volume fraction was estimated separately.

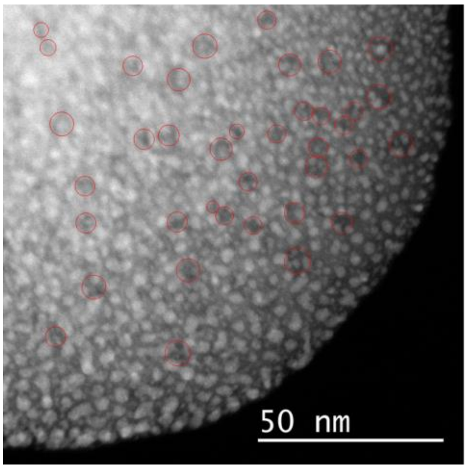

Figure S13. STEM-HAADF image of a nanocomposite TiON/C–CNF2. The red circles are a rough estimate of the size and location of nanopores.

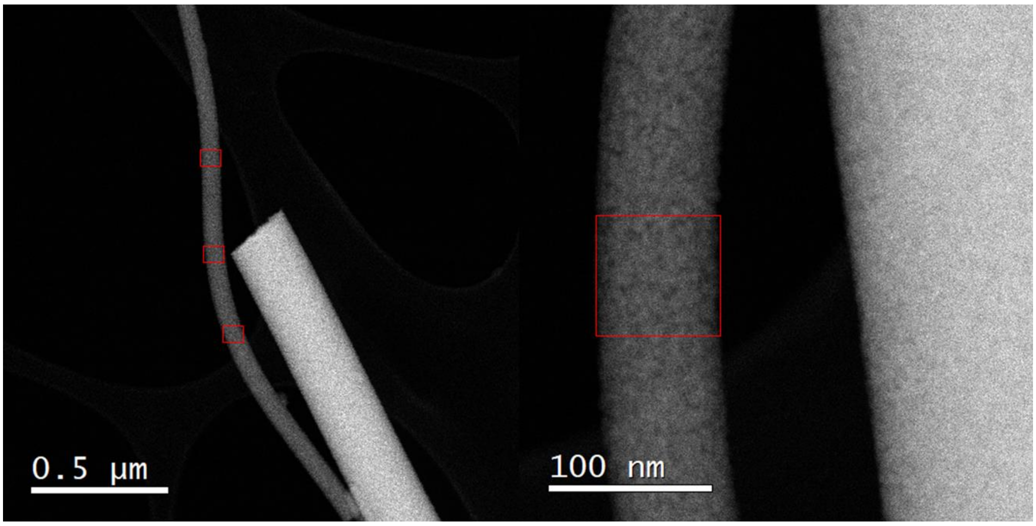

Figure S14. STEM–HAADF image of TiON/C–CNF2s with examples of EELS measurement locations and analysis area size (red square).

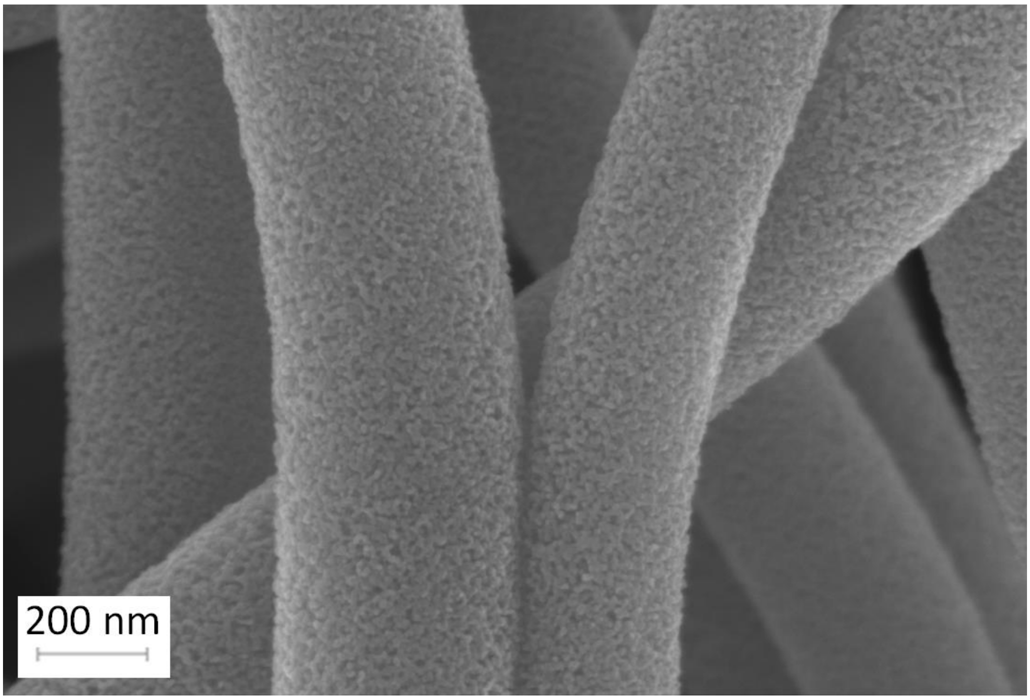

Figure S15. SEM image of TiON/C–CNF1s at high magnification.

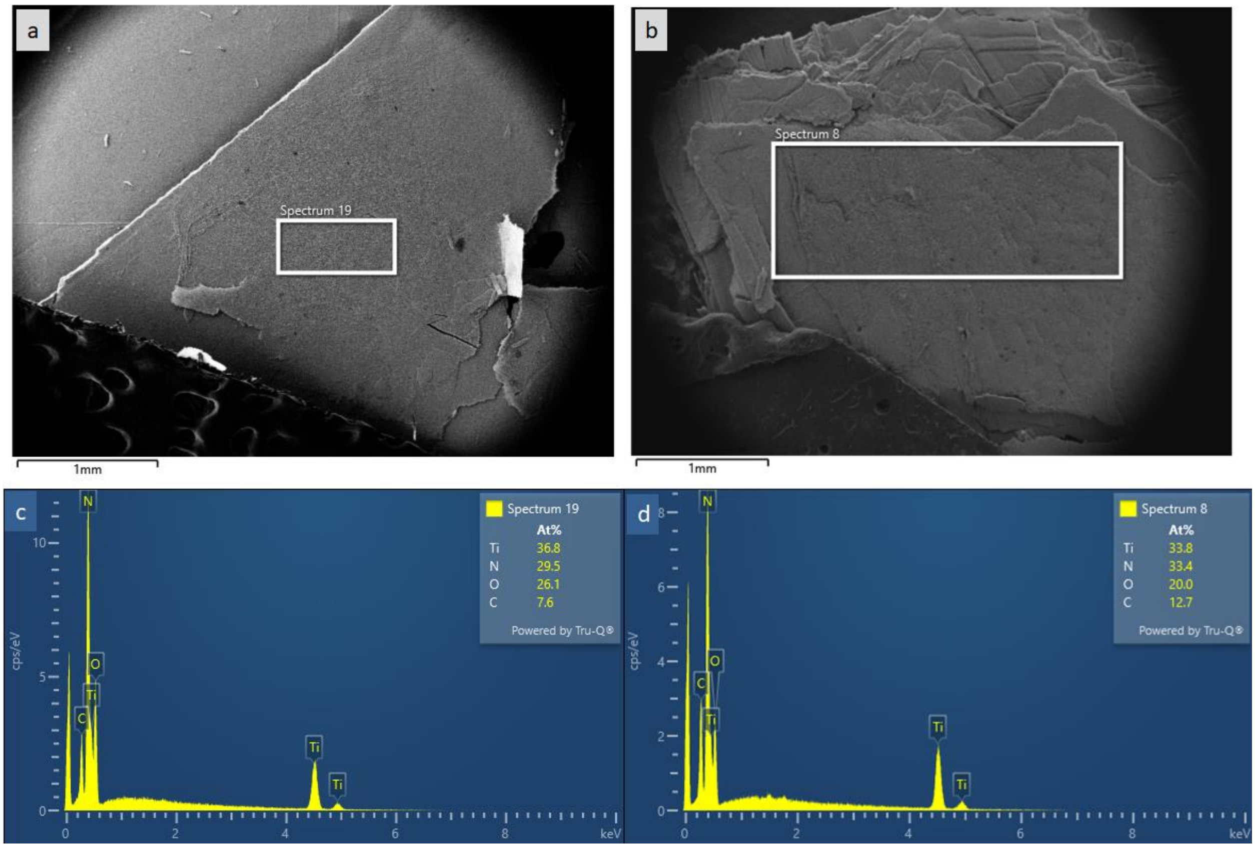

Figure S16. SEM EDXS analysis of TiON/C–CNF1 and TiON/C–CNF2 fabric. (**a**) SEM image of TiON/C–CNF1s where SEM EDXS analysis was made, (**b**) SEM image of TiON/C–CNF2s where SEM EDXS analysis was made, (**c**) SEM EDXS analysis of TiON/C–CNF1, (**d**) SEM EDXS analysis of TiON/C–CNF1.

References

1. Begun, G.M.; Bamberger, C.E. Raman Spectroscopic Observation of Laser-Induced Oxidation of Transition-Metal Borides, Carbides, and Nitrides **2016**, *43*, 134–138. https://doi.org/10.1366/0003702894201914.
2. Doiron, B.; Li, Y.; Mihai, A.; Bower, R.; Alford, N.M.N.; Petrov, P.K.; Maier, S.A.; Oulton, R.F. Plasmon-Enhanced Electron Harvesting in Robust Titanium Nitride Nanostructures. *J. Phys. Chem. C* **2019**, *123*, 18521–18527. https://doi.org/10.1021/ACS.JPCC.9B03184/SUPPL_FILE/JP9B03184_SI_001.PDF.
3. Ohsaka, T.; Izumi, F.; Fujiki, Y. Raman Spectrum of Anatase, TiO2. *J. Raman Spectrosc.* **1978**, *7*, 321–324. https://doi.org/10.1002/JRS.1250070606.
4. Spengler, W.; Kaiser, R. First and Second Order Raman Scattering in Transition Metal Compounds. *Solid State Commun.* **1976**, *18*, 881–884. https://doi.org/10.1016/0038-1098(76)90228-3.
5. Bernard, M.; Deneuville, A.; Thomas, O.; Gergaud, P.; Sandstrom, P.; Birch, J. Raman Spectra of TiN/AlN Superlattices. *Thin Solid Films* **2000**, *380*, 252–255. https://doi.org/10.1016/S0040-6090(00)01531-5.
6. Ding, Z.H.; Yao, B.; Qiu, L.X.; Lv, T.Q. Raman Scattering Investigation of Nanocrystalline δ-TiNx Synthesized by Solid-State Reaction. *J. Alloys Compd.* **2006**, *421*, 247–251. https://doi.org/10.1016/J.JALLCOM.2005.11.017.
7. Ritchie, N.W.M.; Davis, J.; Newbury, D.E. DTSA-II: A New Tool for Simulating and Quantifying EDS Spectra - Application to Difficult Overlaps. *Microsc. Microanal.* **2008**, *14*, 1176–1177, doi:10.1017/S143192760808361X.
